# PReS-FINAL-2276: IgG4 related disease in a 10-year-old girl

**DOI:** 10.1186/1546-0096-11-S2-P266

**Published:** 2013-12-05

**Authors:** S Pasic, G Ristic, S Djuricic

**Affiliations:** 1 Pediatric Immunology, Mother & Child Health Institute, Belgrade, Serbia; 2 Pathology, Mother & Child Health Institute, Belgrade, Serbia

## Introduction

A 10-year-old girl developed an enlargement of parotid and submandibular salivary glands and lymph nodes up to 2.5 cm associated with edema of her upper eyelids. In further follow-up, she developed non-palpable, vasculitic skin lesions. She received cephixime, that co-incided with partial regression of lymph nodes. Re-occurrence of vasculitic lesions with bilateral edema of upper eyelids resembling Miculitz disease was observed. At 7 years of age, she developed asthma requiring the use of bronchodilators. Clinical examination: a 10-year-old girl, pale skin, at the both calves irregularly shaped, livid, painless, vasculitic lesions, 2-3 cm in size. The upper eyelids are swollen. Parotid and submandibular salivary glands and submandibular lymph nodes are enlarged.

## Objectives

To present clinical and laboratory investigations in IgG4-related disease in childhood.

## Methods

Routine laboratory tests including serum Ig concentrations, autoantibody screen, and histopathologic examination.

## Results

Serum proteins were 101 g/l, albumins,38 g/l. Serum immunoglobulin IgA 1.92, IgM 0.38, IgG 42.2 g/l; IgG subclasses: IgG1 18.9 g/l (4.32-10.2), IgG2 17.05 g/l (0.72-4.3), IgG3 6.35 g/l (0.13-0.85), IgG4 9.02 g/l (0.02-0.93). Serum IgE 900 IU/ml (normal up to 60 IU/ml). Coombs test negative. Autoantibody screening was negative. Serologic tests to HSV, EBV, CMV, hepatitis B and C, HIV were negative. Serum ACE was normal, 57 U/l. Neck ultrasound showed an enlargement of both parotid glands, lymph nodes and submandibular salivary glands up to 2.5 cm.

Histopathathologic evaluation of lymph nodes, submandibular and parotid gland: the enclosed lymph node profiles show retained architecture with prominent reactive follicular hyperplasia. The intervening paracortex focally appears prominently hypovascular and contains a polymorphous lymphoid infiltrate comprising small lymphocytes, scattered immunoblasts, some plasma cells and focally prominent eosinophils.The salivary gland tissue shows abundant mononuclear inflammatory infiltrate with focal prominence of plasma cells and significant patchy sclerosis. The immunostainings including CD3, CD20 and CD79a show retained lymph node architecture. There is a reactive pattern of expression of Ki67, CDI0, bcl-2, bCl-G, CD21 and CD23. The immunostains also highlight fragmentation of some of the germinal centres. Most of the IgG staining plasma cells were of IgG4 positive phenotype.

## Conclusion

Bilateral, symmetrical, painless swelling of their lacrimal and salivary (parotid and submandibular) glands should raise suspicion on IgG4-related disease in childhood.

## Disclosure of interest

None declared.

